# Drug interactions with apixaban: A systematic review of the literature and an analysis of VigiBase, the World Health Organization database of spontaneous safety reports

**DOI:** 10.1002/prp2.647

**Published:** 2020-09-02

**Authors:** Silvia Fernandez, Camille Lenoir, Caroline Samer, Victoria Rollason

**Affiliations:** ^1^ Division of Clinical Pharmacology and Toxicology Department of Anesthesiology Pharmacology Intensive Care, and Emergency Medicine Geneva University Hospitals & University of Geneva Geneva Switzerland

**Keywords:** anticoagulants, apixaban, drug interactions, drug safety, pharmacovigilance, systemic review

## Abstract

Apixaban, a direct oral anticoagulant, has emerged over the past few years because it is considered to have a low risk of drug‐drug interactions compared to vitamin K antagonists. To better characterize these interactions, we systematically reviewed studies evaluating the drug‐drug interactions involving apixaban and analyzed the drug‐drug interactions resulting in an adverse drug reaction reported in case reports and VigiBase. We systematically searched Medline, Embase, and Google Scholar up to 20 August 2018 for articles that investigated the occurrence of an adverse drug reaction due to a potential drug interacting with apixaban. Data from VigiBase came from case reports retrieved up to the 2 January 2018, where identification of potential interactions is performed in terms of two drugs, one adverse drug reaction triplet and potential signal detection using Omega, a three‐way measure of disproportionality. We identified 15 studies and 10 case reports. Studies showed significant variations in the area under the curve for apixaban and case reports highlighted an increased risk of hemorrhage or thromboembolic events due to a drug‐drug interaction. From VigiBase, a total of 1617 two drugs and one adverse drug reaction triplet were analyzed. The most reported triplet were apixaban—aspirin—gastrointestinal hemorrhage. Sixty‐seven percent of the drug‐drug interactions reported in VigiBase were not described or understood. In the remaining 34%, the majority were pharmacodynamic drug‐drug interactions. These data suggest that apixaban has significant potential for drug‐drug interactions, either with CYP3A/P‐gp modulators or with drugs that may impair hemostasis. The most described adverse drug reactions were adverse drug reactions related to hemorrhage or thrombosis, mostly through pharmacodynamic interactions. Pharmacokinetic drug‐drug interactions seem to be poorly detected.

## 1 INTRODUCTION

Direct oral anticoagulants (DOACs) act by direct inhibition of coagulation factor II (thrombin) or factor Xa,^1^, ^2^ in contrast with heparin or vitamin K antagonists (VKAs). DOACs have emerged over the past few years from the need for a new generation of oral anticoagulants with a more predictable and safer pharmacological profile and more suitable for long‐term use. They have become an alternative to VKAs, the only drugs available for long‐term anticoagulation for decades.

DOACs have several advantages over other types of anticoagulants: rapid onset and offset of action, a wide therapeutic window and a predictable anticoagulant response that allows fixed doses and eliminates the need for routine monitoring. Moreover, they are considered to be at low risk of drug‐drug interactions (DDIs) and food‐drug interactions compared to VKAs.^2^, ^3^

Concerning safety, DOACs have been associated with a lower risk of intracranial hemorrhage compared to VKAs and to sequential treatment with low‐molecular‐weight heparin (LMWH) and VKAs, regardless of their therapeutic indication.^4^ There is evidence suggesting a lower mortality risk after suffering a major hemorrhage in patients under DOACs than in patients taking VKAs or LMWH‐VKAs,^5^, ^6^ but conversely, DOACs are associated with a higher risk of gastrointestinal hemorrhage.^7^, ^8^

Currently, there are five DOACs approved for use worldwide: an oral direct thrombin inhibitor, dabigatran,^9^ and four oral direct factor Xa inhibitors: rivaroxaban, apixaban, edoxaban, and betrixaban.^10^

Apixaban is used for the prevention of atrial thromboembolic events in patients with nonvalvular atrial fibrillation and venous thromboembolism (VTE) recurrence and prevention in major orthopedic surgery and for the treatment of acute VTE.^11^ In patients with atrial fibrillation (AF), apixaban was superior to warfarin in the prevention of stroke or systemic embolism.^12^ For the treatment of acute VTA, apixaban was noninferior to enoxaparin combined with warfarin.^13^ Overall, the results from the three ADVANCE trials showed a higher efficacy of apixaban than enoxaparin in the prevention of VTE after total hip or knee replacement.^14^, ^15^, ^16^

Small to modest effects in the pharmacokinetic/pharmacodynamic (PK/PD) profile of apixaban were observed in relation to sex and age, thus considered of no clinical relevance. No dose adjustments are therefore recommended for apixaban regarding sex or age alone.^11^, ^17^ Apixaban exposure increased by 30% in the low‐body‐weight group and decreased by 20% in the high body weight group when compared with a reference weight group. The magnitude of these changes was not considered clinically meaningful either, and no dose adjustment based on body weight alone is recommended.^18^ However, a dose reduction is recommended for patients with a body weight < 60 kg and age > 80 years or serum creatinine > 1.5 mg/dL.^11^ Likewise, apixaban exposure was not significantly modified by mild and moderate hepatic impairment (Child‐Pugh A and B, respectively), but apixaban is contraindicated in Child‐Pugh C.^11^

The half‐life of apixaban is 8‐15 h and it is metabolized by cytochrome P450 (CYP) 3A and is a P‐glycoprotein (P‐gp) substrate. Apixaban is therefore at risk of DDIs with CYP3A/P‐gp inhibitors and inducers.^19^, ^20^

The overall objective of this study was to evaluate DDIs involving apixaban by a review of the current published data available in the literature and by a real‐life assessment of the data on apixaban interactions from VigiBase, the WHO (World Health Organization) global database of individual case safety reports (https://www.who‐umc.org).^21^

## 2 MATERIALS AND METHODS

### 2.1 Literature search

To select relevant publications, we applied the eligibility criteria described in Table 1, divided into two main categories as suggested by the Preferred Reporting Items for Systematic Reviews and Meta‐Analyses (PRISMA) statement.^22^ The literature search was conducted in two databases, namely PubMed via MEDLINE and Embase, and in Google Scholar for articles up to the 20th of August 2018.

**TABLE 1:** Eligibility criteria

Study characteristics	Report characteristics
Type of studies In vitro and animal studies Randomized controlled trials Non‐randomized studies Observational studies (including case series and case reports) Type of participants (human studies) Healthy subjects Patients under DOAC therapy for any pathology Type of outcome Effect of potential interacting drugs on PK/PD profile of DOACs Effect of potential interacting drugs on DOACs safety profile: increase in the risk of hemorrhage or thromboembolic events Effects of DOACs on the PK/PD profile of potential interacting drugs	Language of publication English Type of publications Published full‐text articles Congress abstracts Year of publication From database inception to present (PubMed, Embase) From 2011 to present (Google Scholar)

Abbreviations: DOAC: direct oral anticoagulant / PD: pharmacodynamic / PK: pharmacokinetic

The literature search was performed for four DOACs (apixaban, rivaroxaban, dabigatran, and edoxaban) and the search strategy was developed separately for PubMed, Embase, and Google Scholar. For PubMed, keywords/strings were (rivaroxaban OR apixaban OR dagigatran OR edoxaban) OR (DOACs OR NOAC OR « direct oral anticoagulants » OR « new oral anticoagulants » OR « direct thrombin inhibitor » OR « direct factor Xa inhibitor ») AND (drug interaction OR interaction).

In Embase, the keywords/strings used were (rivaroxaban OR apixaban OR dabigatran OR edoxaban) OR (DOACs OR NOAC OR « direct oral anticoagulants » OR « new oral anticoagulants » OR « direct thrombin inhibitor » OR « direct factor Xa inhibitor ») AND drug interaction.

Finally, in Google Scholar, the keywords rivaroxaban OR apixaban OR dabigatran OR edoxaban AND interaction OR interactions AND « case report » were applied.

The reference managing software Zotero® (version 5.0.47) removed duplicates, and two reviewers screened the title and abstract of the remaining records for potential relevance. If more than one article described a single study and each presented the same data, the most recent one was included. Articles were split into two groups: interaction studies and case reports.

The verification process was performed by reviewing the SmPC (Summary of Product Characteristics),^11^ UpToDate‐Lexicomp,^23^ the Table of cytochromes P450 and P‐gp substrates and the table of inhibitors and inducers of cytochromes P450 and P‐gp (https://www.hug‐ge.ch/sites/interhug/files/structures/pharmacologie_et_toxicologie_cliniques/a5_cytochromes_6_2.pdf).^24^ Case reports where the DDIs was not documented or understood from a pharmacological point of view were excluded.

For interaction studies, the types of interactions assessed were PK interactions mediated by CYP3A and P‐gp modulators or gastric pH modifiers and PD interactions mediated by other antithrombotic agents and nonsteroidal anti‐inflammatory drugs (NSAIDs). Interactions not matching any of the previous categories were pooled into an additional category called "other drugs".

Data from these study were classified into in vitro/animal studies or phase I to phase IV human studies. Each study was reviewed and described individually. Moreover, each DDI described in an included study was compared with those described in the SmPC. This post hoc analysis allowed us to assess if some DDI were missing and if the SmPC included all data described in the literature.

For case reports, information collected (when available) was the following: patient characteristics, information on apixaban (dosage, start and end of treatment, duration of treatment) and potential interacting drugs, ADR description, and list of additional medication. A review of the list of potential interacting drugs was then performed by checking the SmPC, UpToDate‐Lexicomp,the table of cytochrome P450 substrates and the table of inhibitors and inducers of cytochrome P450 and P‐gp.^11^, ^23^, ^24^

### 2.2 Analysis of data from spontaneous reports in VigiBase

To explore DDIs between apixaban and other drugs, we used spontaneous reports from VigiBase. VigiBase is maintained by the Uppsala Monitoring Centre (UMC), the WHO Collaborating Centre for International Drug Monitoring. The UMC receives reports of suspected ADRs from national centers in countries participating in the WHO Program for International Drug Monitoring (https://www.who‐umc.org/vigibase/vigibase/). At the date of retrieval (02.01.2018), there were a total of 16,329,758 individual case safety reports in VigiBase for all drugs and all ADRs, and these came from 131 countries. Drugs are coded according to WHODrug and adverse drug reactions (ADR) according to MedDRA (version 20.1). The information in VigiBase comes from a variety of sources, and the probability that the suspected adverse effect is drug‐related is not the same in all cases.^25^

The identification of potential DDIs from Individual Case Safety Report (ICSR) data in VigiBase is performed in terms of drug‐drug‐ADR (DDA) triplets. The analysis of DDA triplets to detect potential signals of DDI is performed using Omega (Ω), an observed‐to expected three‐way measure of disproportionate reporting developed by the UMC.^26^

Ω indicates the frequency of reporting of certain DDA triplets in the dataset compared to what is expected based on the relative reporting in the dataset. A positive Ω indicates an increased risk of the ADR when two drugs are used together compared to the sum of the individual risks when each drug is taken separately.^27^ Therefore, the Ω value may increase or decrease as new reports enter VigiBase. Ω_0,25_ is used as a threshold in the screening of potential DDIs because it is the lower limit of a 95% credibility interval for Ω. Prior to analysis, the dataset was cleaned, first by removing all DDAs with Ω_ 0,25_ less than or equal to 0. Then, some non‐relevant MedDRA preferred terms were excluded, such as “condition aggravated” because they are not real ADRs. Similarly, some non‐relevant drug names were also excluded, such as “placebo” or “drug name/s under assessment for WHO‐DD”. Finally, all rows with drugs reported as “concomitant” were removed from the file, therefore only drugs reported as “interacting” or “suspected” were kept. For analysis of the seriousness and the outcome, each ICSR was summarized to only one line, according to the column with the outcomes. We chose to keep the line with the worst outcome (Fatal > not recovered/not resolved > recovering/resolving > recovered/resolved with sequelae > recovered/resolved > unknown) and seriousness (death > life‐threatening > caused/prolonged hospitalization > disabling/incapacitating > congenital anomaly/birth defect > other).

The search and extraction from VigiBase of ICSRs related to apixaban and DDIs was performed by the UMC on 24 April 2018 from a database freeze conducted on the 2 January 2018.

We considered the number of DDA triplets related to each MedDRA system organ class (SOC), the number of DDA triplets for apixaban and one specific ADR and the number of combinations for apixaban—one specific suspected/interacting drug in the DDA triplet. The data for the outcome and the seriousness were extracted and their number was calculated.

We classified the DDIs as linked to the PK or PD mechanism: PK DDIs were further classified as due to absorption (PKA), distribution (PKD), metabolism (PKM), or excretion (PKE) and PD DDIs according to the direct effect at receptor function (PD1), interference with a biological or physiological control process (PD2) or additive/opposed pharmacological effect (PD3). When a DDI was verified for the two mechanisms, they were counted in both. These DDIs were classified according to SmPC, UpToDate, and PubMed. When more than one mechanism was found, all were listed.

Due to the large quantity of data extracted with the VigiBase analysis, this article focuses on apixaban only.

## 3 RESULTS

### 3.1 Literature

**FIGURE 1:**
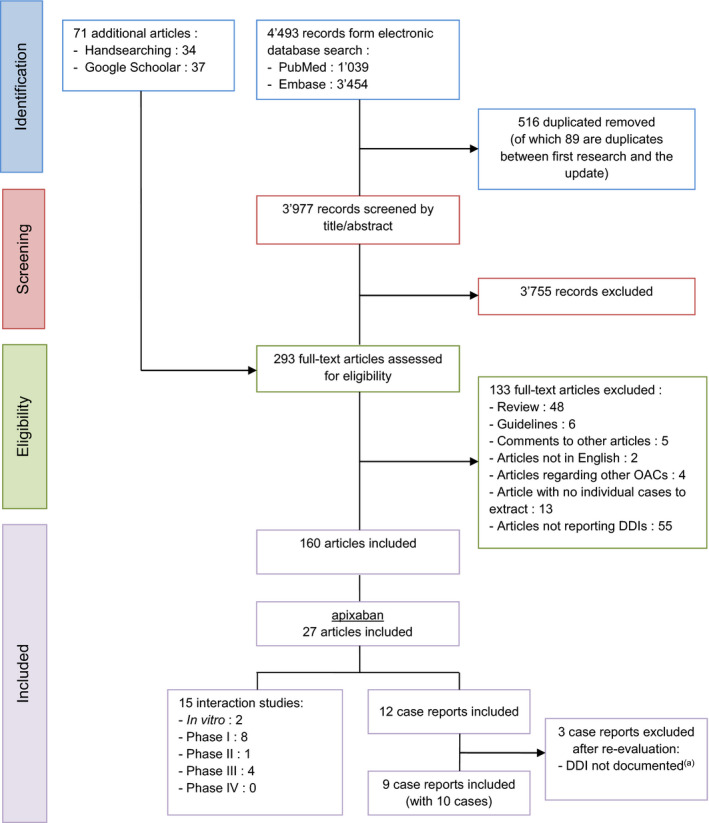
PRISMA flowchart of the apixaban studies selection process

The literature search retrieved 15 interaction studies, some investigating several drugs, and 10 case reports (from nine published articles). The selection process is illustrated in the PRISMA flowchart (Figure 1) and Table 2 summarizes the interaction studies.

**TABLE 2:** Summary of interaction studies involving apixaban

Interaction tested	Reference	Type of study	Effect observed
**CYP3A4/P‐gp inhibitors**			
Ketoconazole	[30]	Phase I	↑ 99% AUC
Diltiazem	[30]	Phase I	↑ 40% AUC
Amiodarone	[33]	Phase III	NS effect
Tacrolimus	[28]	In vitro	No interaction
	[31]	Phase I	NS effect (↓ 22% AUC)
PDE5 (sildenafil, tadalafil, vardenafil)	[29]	In vitro	↓ efflux (97%, 74%, and 100%, respectively)
Cyclosporin	[31]	Phase I	↑ 20% AUC
Clarithromycin	[32]	Phase I	↑ 60% AUC
**CYP3A4/P‐gp inducers**			
Rifampicin	[34]	Phase I	↓ 39% and 54% AUC (iv and oral administration, respectively)
**CYP3A4/P‐gp substrates**			
Digoxin	[35]	Phase I	No effect
**Antithrombotic agents** **and NSAIDs**			
Enoxaparin	[36]	Phase I	↑ anti‐factor Xa activity
Naproxen	[37]	Phase I	↑ 55% AUC
Aspirin	[38]	Phase II	↑ risk of bleeding
	[39]	Phase III	↑ risk of bleeding
	[40]	Phase III	↑ risk of bleeding
	[41]	Phase III	↑ risk of bleeding
Aspirin + clopidogrel	[38]	Phase II	↑ risk of bleeding
	[39]	Phase III	↑ risk of bleeding
	[40]	Phase III	↑ risk of bleeding
**Gastric pH modifiers**			
Famotidine	[42]	Phase I	No effect
**Other drugs**			
AS, CS, HA, klonopin, penicillin, TC, TA	[28]	In vitro	No effect
Atenolol	[35]	Phase I	NCR effect

Abbreviations: AS: alendronate sodium; AUC: area under the plasma concentration‐time curve; CS: chondroitin sulfate; HA: hydrocodone‐acetaminophen; NCR: nonclinically relevant; NS: nonsignificant; TA: tranexamic acid; TC: tramadol chlorhydrate.

#### 3.1.1 CYP3A and P‐gp inhibitors

##### In vitro

In an in vitro study performed by Sayani et al, apixaban did not interact with tacrolimus when combined into citrated plasma.^28^

In another in vitro study performed by Margelidon‐Cozzolino et al, three PDE5 inhibitors (sildenafil, tadalafil, and vardenafil) strongly inhibited apixaban efflux by P‐gp suggesting potential clinically relevant DDI.^29^ The maximal inhibition was higher with vardenafil and sildenafil than with tadalafil.^29^

##### Phase I studies

In healthy volunteers, ketoconazole increased apixaban AUC and Cmax by 2‐fold and 1.6‐fold, respectively.^30^ Likewise, coadministration of apixaban and diltiazem resulted in a 1.4‐fold and 1.3‐fold increase in apixaban AUC and Cmax, respectively.^30^ In healthy volunteers, the administration of ciclosporin led to an increase of 43% and 20% in the Cmax and AUC of apixaban, respectively.^31^ This did not warrant dose modification.^31^ Administration of tacrolimus led to a 13% and a 22% decrease in the Cmax and the AUC of apixaban, respectively, but it did not reach statistical significance.^31^ Finally, administration of clarithromycin to healthy volunteers led to an increase in the Cmax and the AUC of 30% and 60%, respectively, compared to administration of apixaban alone.^32^

##### Phase III studies

Flaker et al analyzed the influence of amiodarone on the outcomes of the ARISTOTLE trial, which compared apixaban and warfarin for the prevention of stroke or systemic embolism in AF patients.^33^ Statistical analysis performed in their study only compared apixaban versus warfarin. Thus, there is no head‐to‐head comparison for each anticoagulant with or without amiodarone. Nevertheless, the observed rates for safety endpoints seem to indicate that, in the ARISTOTLE trial, there were no significant differences concerning the incidence of hemorrhagic events for apixaban with or without amiodarone (eg, the major hemorrhage rate for apixaban with amiodarone is 1.86%/year and without amiodarone is 2.18%/year).^33^

#### 3.1.2 CYP3A and P‐gp inducers

##### Phase I studies

In healthy subjects, rifampicin reduced the AUC of apixaban by 54% and the Cmax by 42%.^34^

#### 3.1.3 CYP3A and P‐gp substrates

##### Phase I studies

The digoxin PK profile was not affected by apixaban co‐administration.^35^

#### 3.1.4 Other antithrombotic agents and NSAIDs

##### Phase I studies

A phase I study carried out by Barrett et al showed that enoxaparin did not modify the PK of apixaban. Nevertheless, enoxaparin was associated with an additive increase in the anti‐factor Xa activity of apixaban.^36^

Combined administration of apixaban and naproxen increased apixaban exposure (54% increase in AUC, 61% increase in Cmax), but led to no clinically relevant prolongation of the bleeding time.^37^

##### Phase II studies

Apixaban was associated with a dose‐dependent increase in clinically relevant hemorrhagic events during the APPRAISE trial, a phase II study in patients with recent acute coronary syndrome (ACS) receiving antiplatelet therapy (aspirin alone or with clopidogrel). This increase was more pronounced in patients receiving dual antiplatelet agents than aspirin alone with apixaban.^38^

##### Phase III studies

In the APPRAISE‐2 trial, coadministration of apixaban with antiplatelet therapy (aspirin alone or aspirin plus clopidogrel) significantly increased major hemorrhagic events, including fatal and intracranial hemorrhages in high‐risk ACS patients. This increase was not associated with a significant decrease in recurrent ischemic events, which is why the trial was terminated prematurely.^39^, ^40^

In AF patients, the concomitant use of aspirin and apixaban or warfarin (ARISTOTLE trial) was associated with a higher hemorrhage risk in both groups. However, a similar benefit/risk profile of apixaban vs warfarin remained regardless of concomitant aspirin use.^41^

#### 3.1.5 Gastric pH modifiers

##### Phase I studies

In healthy subjects, the H_2_ antagonist famotidine had no impact on apixaban's PK.^42^

#### 3.1.6 Other drugs

##### In vitro studies

No DDI was observed when apixaban was supplemented into a citrated plasma combination with the following drugs: alendronate sodium, chondroitin sulfate, hydrocodone‐acetaminophen, klonopin, penicillin, tramadol chlorhydrate, and tranexamic acid.^28^

##### Phase I studies

A study conducted by Frost et al. established that there is no clinically relevant DDI between apixaban and atenolol. The co‐administration of both drugs led to a slight decrease in apixaban exposure (15% decrease in AUC and 18% decrease in Cmax).^35^

### 3.2 Case reports

Ten case reports in nine publications relating to apixaban were found in the literature.^43^, ^44^, ^45^, ^46^, ^47^, ^48^, ^49^, ^50^, ^51^ Cases concerned mainly men except for three cases, and the age range was 43‐88 years old. Apixaban indication was AF in all cases. Additional pathophysiological factors contributing to the development of the ADR were reported in several cases, the most relevant being renal impairment.

With regard to the mechanism of DDI, five cases were PK interactions, three cases were PD interaction, and two involved both PK and PD interactions. Concerning the PK interaction, two cases were treated with CYP3A and/or P‐gp inhibitors and three cases were treated with P‐gp and/or CYP3A inducers. For CYP3A/P‐gp inhibitors, both case led to a hemorrhage, but one case involved an interaction with diltiazem and the second involved an interaction with diltiazem and amiodarone.^43^, ^44^ For the CYP3A and/or P‐gp inducers, in the first case, an interaction with carbamazepine was deemed possible, but the apixaban concentrations were still lower than expected after discontinuation of carbamazepine.^47^ In another case, apixaban plasma concentration increased fourfold (89 ng/mL to 361 ng/mL) after phenobarbital discontinuation.^51^ In the last case of induction, the co‐administration of efavirenz with apixaban led to a pulmonary embolism.^50^ Two case reports described cardiac tamponade after apixaban and ibrutinib co‐administration, caused by a PD interaction.^48^, ^49^ The last PD interaction involved an SSRI alone.^46^ For the PK/PD interactions, one case involved a selective serotonin reuptake inhibitor (SSRI) that induced platelet dysfunction and CYP34 inhibition (^45^) and one case involved both an SSRI (platelet dysfunction) and a CYP3A/P‐gp inhibitor.^46^

### 3.3 VigiBase

A total of 1654 DDA triplets with positive _0,25_ values were extracted from VigiBase for the DDA triplet combination apixaban—any suspected/interacting drug—any ADR. These DDA triplets came from 3137 ICSRs reported to VigiBase up to the database freeze conducted in January 2018.

After the cleaning of the dataset, 1617 DDA triplets (corresponding to 263 unique DDA triplet combinations with apixaban—one specific suspected/interacting drug—one defined ADR, each observed in several ICSRs) and 1'364 ICSRs remained for analysis.

The MedDRA SOCs most represented in the dataset were "GI disorders" (30.5%, n = 493), "investigations" (9.5%, n = 153), "respiratory, thoracic, and mediastinal disorders" (8.2%, n = 133), and "cardiac disorders" (8.0%, n = 130). The three most reported ADRs at a MedDRA PT level in combination with apixaban and any other suspected/interacting drug were GI hemorrhage (22.7%, n = 367), decreased hemoglobin (5.1%, n = 82), and AF (4.0%, n = 64). Irrespective of the ADR, the three suspected/interacting drugs that were the most co‐reported with apixaban were acetylsalicylic acid (ASA) (27,6%, n = 446), rivaroxaban (10.9%, n = 176), and clopidogrel (5.7%, n = 92). If the ADRs reported for each of those drug pairs were also considered separately, the ADR the most reported for the pair apixaban and ASA was GI hemorrhage (49.6%, n = 221), that for apixaban plus rivaroxaban was also GI hemorrhage (58.0%, n = 102) and that for the pair apixaban‐clopidogrel was decreased hemoglobin (23.9%, n = 22).

The three most reported DDA triplets in the whole dataset were as follows: apixaban‐ASA‐GI hemorrhage (13.7%. n = 221), apixaban‐rivaroxaban‐GI hemorrhage (6.3%, n = 102), and apixaban‐ASA‐decreased hemoglobin (2.5%, n = 40).

**FIGURE 2:**
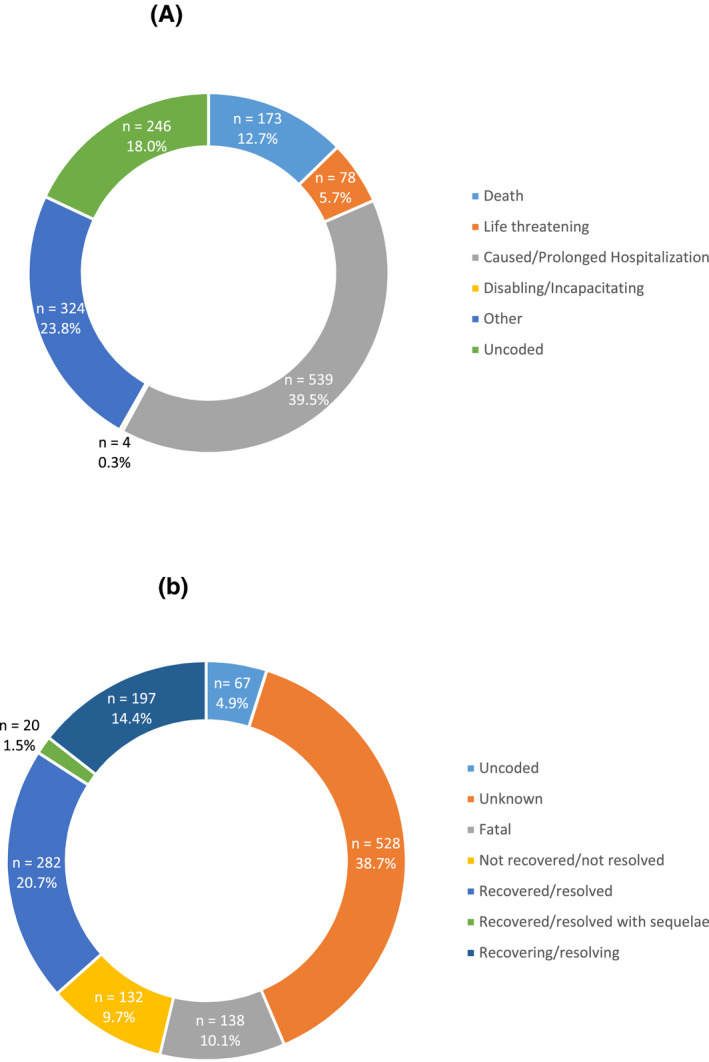
Summary of the results on the seriousness and the outcomes reported in the ICSRs extracted from VigiBase. A, Seriousness B, Outcome

Not all ICSRs had data regarding the seriousness and outcome. In 12.2% (n = 246) and in 4.9% (n = 67) of the ICSRs, information about the seriousness and outcome was not filled in. Figure 2 shows the results of the analysis of the data on the seriousness and the outcome reported in the ICSRs (n = 1365).

Figure 2A shows the different seriousness reported and their proportions. In slightly more than a third of the ICSRs (39.5%, n = 539), the ADR was reported as caused/prolonged hospitalization. The ADR led to the patient's death in 12.7% (n = 173) of the ICSRs and was life threatening in 5.7% (n = 78). It was reported as disabling/incapacitating in only four cases (0.3%). In 23.8% (n = 324) of the cases, the seriousness was reported as "other" (those belonging to none of the other categories) (Figure 2A).

As illustrated in Figure 2B, the outcome was unknown in a large proportion of the ICSRs (38.7%. n = 528). Ten percent of the cases (10.1%, n = 138) had a fatal outcome. The patients recovered in 22.1% (n = 302) of cases (1.5%, n = 20, with sequelae and 20.7%, n = 282, without sequelae), whereas in 9.7% (n = 132) of the ICSRs the patient did not recover from the ADR. A total of 14.4% (n = 197) of the patients were deemed as recovering when the case was reported to VigiBase (Figure 2B).

Of the 263 DDA triplets reviewed, 179 DDIs were not described in the literature. For the others, a total of 12 PK DDIs, 68 PD DDIs, and 4 PK/PD DDIs were described in the literature. The most common PK DDIs was inhibition of drug metabolism, and the most common PD DDIs was additive pharmacological effect. Regarding PK DDIs, inhibitors of CYP3A and P‐gp were the most reported drugs, and hemorrhagic events were the most reported ADR (Table 3). For PD DDIs, antithrombotic agents and NSAIDs were the most reported drugs, and hemorrhage was the most reported ADR. Regarding hemorrhage, the most reported site was gastro‐intestinal hemorrhage (Table 3).

**TABLE 3:** Drug reported as interacting with apixaban in VigiBase with interaction mechanism and most frequently reported adverse effect

Drug B	No. of occurrence	Mechanism	Mechanism sub‐classification	Most frequently reported ADRs (No. observed in parenthesis)
Acenocoumarol	1	PD	Additive pharmacological effect	Anemia (3)
Acetysalicylic acid	18	PD	Additive pharmacological effect	Gastrointestinal disorder (221)
Allopurinol	1	PD	Additive pharmacological effect	Melena (3)
Amiodarone	4	PK	Drug Metabolism	Hemorrhagic anaemia (7)
Celecoxib	1	PD	Additive pharmacological effect	Gastrointestinal hemorrhage (10)
Cilostazol	1	PD PK	‐ Additive pharmacological effect ‐ Drug Metabolism	Cerebral hemorrhage (5)
Citalopram	1	PD	Additive pharmacological effect	Hematuria (3)
Clopidogrel	11	PD	Additive pharmacological effect	Hemoglobin decreased (22)
Dabigatran	1	PD	Additive pharmacological effect	Internal hemorrhage (3)
Diclofenac	2	PD	Additive pharmacological effect	Gastric ulcer hemorrhage (3) Epistaxis (3)
Diltiazem	1	PK	Drug Metabolism	Epistaxis (7)
Dronedarone	1	PK	Drug Metabolism	Transient ischemic attack (3)
Enoxaparin	3	PD	Additive pharmacological effect	Postprocedural hemorrhage (6)
Enzalutamide	1	PK	Drug Metabolism	Hematuria (3)
Fluconazole	2	PK	Drug Metabolism	‐ Hemorrhage intracranial (3) ‐ Hematoma (3)
Heparin	3	PD	Additive pharmacological effect	Muscle hemorrhage (3)
Ibrutinib	7	PD	Additive pharmacological effect	Contusion (13)
Ibuprofen	4	PD	Additive pharmacological effect	Gastrointestinal hemorrhage (11)
Indometacin	1	PD	Additive pharmacological effect	Gastrointestinal disorders (4)
Loxoprofen	1	PD	Additive pharmacological effect	Gastrointestinal hemorrhage (4)
Naproxen	4 3	PD PK	‐ Additive pharmacological effect ‐ Drug Metabolism	Gastrointestinal hemorrhage (9)
Phenprocoumon	2	PD	Additive pharmacological effect	Epistaxis (10)
Prednisolone	1	PD	Additive pharmacological effect	Hemorrhage subcutaneous (3)
Ranolazine	1	PK	Drug Metabolism	Hemorrhage (3)
Rivaroxaban	5	PD	Direct effect at receptor level	Gastrointestinal disorder (102)
Ticagrelor	1	PD	Additive pharmacological effect	Epistaxis (4)
Verapamil	2	PK	Drug Metabolism	Melena (3)
Warfarin	2	PD	Additive pharmacological effect	Contusion (35)

## 4 DISCUSSION

The arrival of apixaban into routine clinical practice was a major step in anticoagulation therapy due to its alleged favorable profile, which translates into undeniable benefits for patients, especially regarding its ease of use. One of the most relevant aspects of apixaban is its theoretically low potential for interactions with other medications, food, and herbal products. However, phase IV or postmarketing studies are necessary to identify further potential DDIs, as apixaban is now used in real‐world situations. To this end, we performed a literature review of published studies and case reports, together with an analysis of data reported to VigiBase. A vast majority of DDIs identified in our literature search, in both interaction studies and case reports, were DDIs with CYP3/P‐gp inhibitors and other antithrombotic agents/NSAIDs. Only a few interaction studies tested the impact of CYP3A and P‐gp inducers, as already pointed out in other reviews.^52^, ^53^ To verify the coverage of our literature search, we performed a post hoc comparison between our collected data and the data contained in the apixaban SmPC elaborated by the European Medicine Agency.^11^ Two DDI studies described in the SmPC were not detected by our literature search, namely, a study with prasugrel and another one with the clopidogrel‐ASA combination. These seem to be unpublished and not registered either in clinicaltrials.gov. Phase I studies in healthy volunteers are not subject to mandatory data disclosure,^54^, ^55^ and their publication depends on the transparency policies of drug manufacturers. A recent study has shown a significantly lower level of transparency for phase I (healthy volunteers) studies compared to studies performed in patients.^55^ Conversely, in vitro interaction studies with tacrolimus and alendronate sodium, chondroitin sulfate, hydrocodone‐acetaminophen, klonopin, penicillin, tramadol chlorhydrate, and tranexamic acid identified in our review, were not mentioned in EMA SmPC because these studies showed the absence of a DDI.^11^ Indeed, in vitro data are only included in the SmPC if they lead to a change in the use of the medicinal product.^56^ Likewise, data from phase IV studies are only included in SmPC if they result in modification of the drug's marketing authorization.^57^ Regarding in vivo data, an absence of interaction should only be mentioned in the SmPC if it is of major importance to the prescriber.^58^ That may explain the absence of information on several phase I, II, and III studies showing nonsignificant or nonclinically relevant interactions. Some of the potential interacting drugs identified in the included case reports were also not mentioned in apixaban SmPC,^11^ such as venlafaxine.

We also compared the ADRs reported in the case reports included in our literature search with those reported in apixaban's SmPC.^11^ Hemopericardium and gluteal artery hemorrhage were identified in our case reports but were not specifically mentioned in apixaban SmPC. However, since data from case reports alone do often not allow to establish causal relationships, further investigation would be needed to confirm these findings.^59^ This is particularly true for DDIs where other factors may have also contributed to the ADR described in the case report.^60^ Considering all the above, it should be underscored that our literature search has some limitations. We searched only for published articles, and thus, we did not retrieve data on unpublished interactions. Moreover, the in vitro data detected may not translate into a clinically relevant interaction in vivo.

Regarding data from VigiBase, the most co‐reported suspecting/interacting drug was ASA, the most co‐reported ADR was GI hemorrhage and, consequently, apixaban‐ASA‐GI hemorrhage was the most reported DDA triplet. DOACs have been associated with an increased risk of GI hemorrhage in multiple studies, including an evaluation of their safety profile based on data from VigiBase.^7^, ^8^ However, this phenomenon has been mainly observed with dabigatran and rivaroxaban and not with apixaban.^7^, ^8^ In the analysis from VigiBase performed by Monaco et al, apixaban was mostly associated with cerebrovascular accident,^8^ an ADR not identified in our interaction dataset. Instead, our dataset included other related terms, such as ischemic stroke, transient ischemic attack or hemorrhagic cerebral infarction, although to a much lesser extent than GI hemorrhage.

Several suspected/interacting drugs were excluded from our analysis of the ICSRs, as they were not documented or understood from a pharmacological perspective as associated with DDIs with DOACs. Additionally, in many DDA triplets, the reported ADR did not seem to correlate with the drug pair, irrespective of whether the drug pair did or did not have an established DDI, such as apixaban‐tamsulosin‐memory impairment or apixaban‐dofetilide‐thirst.

We found that the proportion of PD DDIs was higher than the proportion of PK DDIs, suggesting that apixaban might be at higher‐risk of interacting with drugs with the same pharmacological profile than with CYP3A4/P‐gp inhibitors or inducers. However, this may be a bias, as VigiBase is a database that is dependent on spontaneous ADR reports, and healthcare professionals often know better of PD DDIs. In a study that used this same database, there were more PD DDIs (41%) than PK DDIs (25%).^61^

ADR reporting databases, such as VigiBase, have inherent limitations. The two first limitations to mention are underreporting and selective reporting. Another limitation in these databases is the lack of a denominator that allows estimating a risk. Additionally, the available dataset did not allow us to find a plausible explanation for the DDIs. They could be attributed to the heterogeneity of the data stored in VigiBase, which comes from regulatory and voluntary sources and, in some cases, may lack a proper causality assessment, since not all national pharmacovigilance centers contributing to VigiBase perform causality assessments of their ICSRs.^62^ Finally, the quality and information contained in an ICSR is limited by the way this ICSR was coded into the database, with crucial data, such as the start or stop date of the drug, often missing. Information available in free text in original reports would also be important because it often contains additional relevant clinical details.^63^ This approach entails a detailed case‐by‐case analysis of ICSRs and largely depends on the completeness of each report because it relies on fields that are not mandatory to be fulfilled for reports to be accepted in VigiBase.^64^ To improve drug interaction surveillance in VigiBase, the UMC suggests the use of certain reporting patterns as indicators of DDIs in addition to a positive Ω_0,25_.^65^ Other information useful in identifying suspected adverse drug interactions from ICSRs would be a plausible time course, a positive dechallenge and alternative causes of the reaction.^63^ Our results have to be interpreted in this light.

## 5 CONCLUSION

Our analysis shows that apixaban has significant potential for DDIs with other drugs, mostly CYP3A/P‐gp inhibitors, CYP3A/P‐gp inducers and drugs that may impair hemostasis, such as ASA and NSAIDs, and therefore, a significant number of DDIs with apixaban must be considered by clinicians and patients.

This review of the literature, especially the analysis of reports from VigiBase, notes that pharmacodynamic interactions that occur through the known properties of the drug and that are predictable are widely known and reported. On the other hand, the data analysis shows that the detection and reporting of pharmacokinetic interactions that occur through cytochromes or transporters are sparse because they are badly recognized.

This should motivate clinicians to stay alert on every adverse drug reaction encountered in a patient and to always consider that this adverse drug reaction could also be due to a drug‐drug interaction and can be at least partly avoidable.

## CONFLICT OF INTEREST

The authors declare that they have no conflict of interest.

## Data Availability

The data that support the findings of this study are available from the corresponding author upon reasonable request.

## References

[1] Alquwaizani M , Buckley L , Adams C , Fanikos J . Anticoagulants: a review of the pharmacology, dosing, and complications. Curr Emerg Hosp Med Rep. 2013;1(2):83‐97. 23687625 10.1007/s40138-013-0014-6 PMC3654192

[2] Harter K , Levine M , Henderson SO . Anticoagulation drug therapy: a review. West J Emerg Med. 2015;16(1):11‐17. 25671002 10.5811/westjem.2014.12.22933 PMC4307693

[3] Bauer KA . Pros and cons of new oral anticoagulants. Hematol Am Soc Hematol Educ Program. 2013;2013:464‐470. 10.1182/asheducation-2013.1.464 24319220

[4] Caldeira D , Barra M , Pinto FJ , Ferreira JJ , Costa J . Intracranial hemorrhage risk with the new oral anticoagulants: a systematic review and meta‐analysis. J Neurol. 2015;262(3):516‐522. 25119841 10.1007/s00415-014-7462-0

[5] Skaistis J , Tagami T . Risk of fatal bleeding in episodes of major bleeding with new oral anticoagulants and vitamin K antagonists: a systematic review and meta‐analysis. PLoS One. 2015;10(9):e0137444. 26383245 10.1371/journal.pone.0137444 PMC4575170

[6] Caldeira D , Rodrigues FB , Barra M , et al. Non‐vitamin K antagonist oral anticoagulants and major bleeding‐related fatality in patients with atrial fibrillation and venous thromboembolism: a systematic review and meta‐analysis. Heart Br Card Soc. 2015;101(15):1204‐1211. 10.1136/heartjnl-2015-307489 26037103

[7] Holster IL , Valkhoff VE , Kuipers EJ , Tjwa ETTL . New oral anticoagulants increase risk for gastrointestinal bleeding: a systematic review and meta‐analysis. Gastroenterology. 2013;145(1):105‐112.e15. 23470618 10.1053/j.gastro.2013.02.041

[8] Monaco L , Biagi C , Conti V , et al. Safety profile of the direct oral anticoagulants: an analysis of the WHO database of adverse drug reactions. Br J Clin Pharmacol. 2017;83(7):1532‐1543. 28071818 10.1111/bcp.13234 PMC5465343

[9] Lixiana, INN‐edoxaban ‐ lixiana‐epar‐product‐information_en.pdf. https://www.ema.europa.eu/documents/product‐information/lixiana‐epar‐product‐information_en.pdf Accessed October 25, 2018.

[10] Lippi G , Gosselin R , Favaloro EJ . Current and emerging direct oral anticoagulants: state‐of‐the‐art. Semin Thromb Hemost. 2019;45(5):490‐501. 31216588 10.1055/s-0039-1692703

[11] Eliquis, INN‐apixaban ‐ eliquis‐epar‐product‐information_en.pdf. https://www.ema.europa.eu/documents/product‐information/eliquis‐epar‐product‐information_en.pdf. Accessed October 25, 2018.

[12] Granger CB , Alexander JH , McMurray JJV , et al. Apixaban versus warfarin in patients with atrial fibrillation. N Engl J Med. 2011;365(11):981‐992. 21870978 10.1056/NEJMoa1107039

[13] Agnelli G , Buller HR , Cohen A , et al. Oral apixaban for the treatment of acute venous thromboembolism. N Engl J Med. 2013;369(9):799‐808. 23808982 10.1056/NEJMoa1302507

[14] Lassen MR , Raskob GE , Gallus A , Pineo G , Chen D , Portman RJ . Apixaban or enoxaparin for thromboprophylaxis after knee replacement. N Engl J Med. 2009;361(6):594‐604. 19657123 10.1056/NEJMoa0810773

[15] Lassen MR , Raskob GE , Gallus A , et al. Apixaban versus enoxaparin for thromboprophylaxis after knee replacement (ADVANCE‐2): a randomised double‐blind trial. Lancet Lond Engl. 2010;375(9717):807‐815. 10.1016/S0140-6736(09)62125-5 20206776

[16] Lassen MR , Gallus A , Raskob GE , et al. Apixaban versus enoxaparin for thromboprophylaxis after hip replacement. N Engl J Med. 2010;363(26):2487‐2498. 21175312 10.1056/NEJMoa1006885

[17] Frost CE , Song Y , Shenker A , et al. Effects of age and sex on the single‐dose pharmacokinetics and pharmacodynamics of apixaban. Clin Pharmacokinet. 2015;54(6):651‐662. 25573421 10.1007/s40262-014-0228-0 PMC4449375

[18] Upreti VV , Wang J , Barrett YC , et al. Effect of extremes of body weight on the pharmacokinetics, pharmacodynamics, safety and tolerability of apixaban in healthy subjects. Br J Clin Pharmacol. 2013;76(6):908‐916. 23488672 10.1111/bcp.12114 PMC3845314

[19] Heidbuchel H , Verhamme P , Alings M , et al. Updated European Heart Rhythm Association Practical Guide on the use of non‐vitamin K antagonist anticoagulants in patients with non‐valvular atrial fibrillation. Eur Eur Pacing Arrhythm Card Electrophysiol J Work Groups Card Pacing Arrhythm Card Cell Electrophysiol Eur Soc Cardiol. 2015;17(10):1467‐1507. 10.1093/europace/euv309 26324838

[20] Hellwig T , Gulseth M . Pharmacokinetic and pharmacodynamic drug interactions with new oral anticoagulants: what do they mean for patients with atrial fibrillation? Ann Pharmacother. 2013;47(11):1478‐1487. 24259602 10.1177/1060028013504741

[21] Hugman B . From the uppsala monitoring centre : a review of viewpoint part 1 and part 2. Drug Saf. 2005;28(7):645‐646. 10.2165/00002018-200528070-00006 15963009

[22] Liberati A , Altman DG , Tetzlaff J , et al. The PRISMA statement for reporting systematic reviews and meta‐analyses of studies that evaluate health care interventions: explanation and elaboration. PLoS Med. 2009;6(7):e1000100. 19621070 10.1371/journal.pmed.1000100 PMC2707010

[23] Linking Policy | UpToDate. https://www.uptodate.com/home/linking‐policy. Accessed October 25, 2018.

[24] Samer CF , Lorenzini KI , Rollason V , Daali Y , Desmeules JA . Applications of CYP450 testing in the clinical setting. Mol Diagn Ther. 2013;17(3):165‐184. 23588782 10.1007/s40291-013-0028-5 PMC3663206

[25] Uppsala Monitoring Center, Who Collaborating Centre for International Drug Monitoring. Caveat document. Statement of reservations, limitations and conditions relating to data released from VigiBase, the WHO global database of individual case safety reports (ICSRs). 2018 https://www.who‐umc.org/media/164610/umc_caveat.pdf.

[26] Norén GN , Sundberg R , Bate A , Edwards IR . A statistical methodology for drug‐drug interaction surveillance. Stat Med. 2008;27(16):3057‐3070. 18344185 10.1002/sim.3247

[27] Strandell J . Drug interaction surveillance using individual case safety reports. 2011 http://liu.diva‐portal.org/smash/get/diva2:439241/FULLTEXT01.pdf.

[28] Sayani S , Iqbal O , Hoppensteadt D , Fareed J . Drug interactions of newer oral anticoagulants dabigatran, rivaroxaban, and apixaban with routinely used nonanticoagulant/antiplatelet drugs. Blood. 2014;124(21):4267.

[29] Margelidon‐Cozzolino V , Hodin S , Jacqueroux E , Delézay O , Bertoletti L , Delavenne X . In vitro assessment of pharmacokinetic drug‐drug interactions of direct oral anticoagulants: type 5‐phosphodiesterase inhibitors are inhibitors of rivaroxaban and apixaban efflux by P‐glycoprotein. J Pharmacol Exp Ther. 2018;365(3):519‐525. 29572341 10.1124/jpet.117.245993

[30] Frost CE , Byon W , Song Y , et al. Effect of ketoconazole and diltiazem on the pharmacokinetics of apixaban, an oral direct factor Xa inhibitor. Br J Clin Pharmacol. 2015;79(5):838‐846. 25377242 10.1111/bcp.12541 PMC4415720

[31] Bashir B , Stickle DF , Chervoneva I , Kraft WK . Drug‐drug interaction study of Apixaban with cyclosporine and Tacrolimus in healthy volunteers. Clin Transl Sci. 2018;11(6):590‐596. 29972633 10.1111/cts.12580 PMC6226116

[32] Garonzik S , Byon W , Myers E , Li X , Marchisin D , Murthy B . The effects of Clarithromycin on the pharmacokinetics of apixaban in healthy volunteers: a single‐sequence crossover study. Am J Cardiovasc Drugs. 2019;19(6):561‐567. 31030414 10.1007/s40256-019-00348-2 PMC6885504

[33] Flaker G , Lopes RD , Hylek E , et al. Amiodarone, anticoagulation, and clinical events in patients with atrial fibrillation: insights from the ARISTOTLE trial. J Am Coll Cardiol. 2014;64(15):1541‐1550. 25301455 10.1016/j.jacc.2014.07.967

[34] Vakkalagadda B , Frost C , Byon W , et al. Effect of rifampin on the pharmacokinetics of Apixaban, an oral direct inhibitor of factor Xa. Am J Cardiovasc Drugs Drugs Devices Interv. 2016;16(2):119‐127. 10.1007/s40256-015-0157-9 26749408

[35] Frost C , Song Y , Yu Z , et al. The effect of apixaban on the pharmacokinetics of digoxin and atenolol in healthy subjects. Clin Pharmacol Adv Appl. 2017;9:19‐28. 10.2147/CPAA.S115687 PMC5327911 28260951

[36] Barrett YU , Wang J , Song Y , et al. A randomised assessment of the pharmacokinetic, pharmacodynamic and safety interaction between apixaban and enoxaparin in healthy subjects. Thromb Haemost. 2012;107(5):916‐924. 22398784 10.1160/TH11-09-0634

[37] Frost C , Shenker A , Gandhi MD , et al. Evaluation of the effect of naproxen on the pharmacokinetics and pharmacodynamics of apixaban. Br J Clin Pharmacol. 2014;78(4):877‐885. 24697979 10.1111/bcp.12393 PMC4239981

[38] APPRAISE Steering Committee and Investigators , Alexander JH , Becker RC , et al. Apixaban, an oral, direct, selective factor Xa inhibitor, in combination with antiplatelet therapy after acute coronary syndrome: results of the Apixaban for Prevention of Acute Ischemic and Safety Events (APPRAISE) trial. Circulation. 2009;119(22):2877‐2885. 19470889 10.1161/CIRCULATIONAHA.108.832139

[39] Alexander JH , Lopes RD , James S , et al. Apixaban with antiplatelet therapy after acute coronary syndrome. N Engl J Med. 2011;365(8):699‐708. 21780946 10.1056/NEJMoa1105819

[40] Hess CN , James S , Lopes RD , et al. Apixaban plus mono versus dual antiplatelet therapy in acute coronary syndromes: insights from the APPRAISE‐2 trial. J Am Coll Cardiol. 2015;66(7):777‐787. 26271059 10.1016/j.jacc.2015.06.027

[41] Alexander JH , Lopes RD , Thomas L , et al. Apixaban vs. warfarin with concomitant aspirin in patients with atrial fibrillation: insights from the ARISTOTLE trial. Eur Heart J. 2014;35(4):224‐232. 24144788 10.1093/eurheartj/eht445

[42] Upreti V , Song Y , Wang Y , et al. Effect of famotidine on the pharmacokinetics of apixaban, an oral direct factor Xa inhibitor. Clin Pharmacol Adv Appl. 2013;5:59‐66. 10.2147/CPAA.S41999 PMC3637032 23637566

[43] Aktas H , Inci S , Dogan P , Izgu I . Spontaneous rectus sheath hematoma in a patient treated with apixaban. Intractable Rare Dis Res. 2016;5(1):47‐49. 26989650 10.5582/irdr.2015.01039 PMC4761585

[44] Denetclaw TH , Tam J , Arias V , Kim R , Martin C . Case report: apixaban‐associated gluteal artery extravasation reversed with PCC3 without FFP. J Pharm Pract. 2016;29(4):427‐430. 26519251 10.1177/0897190015613231

[45] Sigawy C , Apter S , Vine J , Grossman E . Spontaneous hemopericardium in a patient receiving Apixaban therapy: first case report. Pharmacotherapy. 2015;35(7):e115–e117. 10.1002/phar.1602 26095120

[46] Sablani N , Garg J , Hasan B , Patel R , Martinez MW . First reported case series in the United States of hemopericardium in patients on apixaban. Hear Case Rep. 2018;4(2):82‐84. 10.1016/j.hrcr.2017.11.015 PMC5988469 29876295

[47] Evanger N , Szkotak A , Stang L , Bungard TJ . Apixaban concentration with and without coadministration of carbamazepine: a case with no apparent interaction. Can J Hosp Pharm. 2017;70(6):463‐467. 29299007 10.4212/cjhp.v70i6.1714 PMC5737190

[48] Nassif T , Banchs J , Yusuf SW , Mouhayar E . Acute haemorrhagic tamponade in cancer patients receiving direct oral anticoagulant: case series. Eur Heart J ‐ Case Rep. 2017;1(2):1‐4. 10.1093/ehjcr/ytx018 PMC6177071 31020076

[49] Khalid M , Khattak F , Ramu V . Cardiovascular side effects of tyrosine kinase inhibitor Ibrutinib (Imbruvica) and interaction with direct oral anticoagulant. Am J Ther. 2018;25(6):e768‐e769. 29916856 10.1097/MJT.0000000000000775

[50] Aponte J , Salonia J , Stoever J , Shujaat A . When Not to Choose Apixaban: A Case of Apixaban Failure. B47. CRITICAL CARE CASE REPORTS: CARDIOVASCULAR DISEASES AND ECHOCARDIOGRAPHY. American Thoracic Society International Conference Abstracts. American Thoracic Society. 2018:A3451–A3451. 10.1164/ajrccm-conference.2018.197.1_MeetingAbstracts.A3451

[51] King PK , Stump TA , Walkama AM , Ash BM , Bowling SM . Management of phenobarbital and Apixaban interaction in recurrent Cardioembolic stroke. Ann Pharmacother. 2018;52(6):605‐606. 29457494 10.1177/1060028018759938

[52] Vazquez SR . Drug‐drug interactions in an era of multiple anticoagulants: a focus on clinically relevant drug interactions. Hematol Am Soc Hematol Educ Program. 2018;2018(1):339‐347. 10.1182/asheducation-2018.1.339 PMC6246002 30504330

[53] Vranckx P , Valgimigli M , Heidbuchel H . The significance of drug‐drug and drug‐food interactions of oral anticoagulation. Arrhythmia Electrophysiol Rev. 2018;7(1):55‐61. 10.15420/aer.2017.50.1 PMC5889806 29636974

[54] Home ‐ ClinicalTrials.gov. https://clinicaltrials.gov/. Accessed October 25, 2018.

[55] Miller JE , Wilenzick M , Ritcey N , Ross JS , Mello MM . Measuring clinical trial transparency: an empirical analysis of newly approved drugs and large pharmaceutical companies. BMJ Open. 2017;7(12):e017917. 10.1136/bmjopen-2017-017917 PMC5728266 29208616

[56] Microsoft Word ‐ SPC GL final 28 10 05.doc ‐ spcguidrev1‐oct2005_en.pdf. http://www.kardio.hr/wp‐content/uploads/2012/12/spcguidrev1‐oct2005_en.pdf. Accessed October 25, 2018.

[57] Guideline on good pharmacovigilance practices (GVP) ‐ Module VIII – Post‐authorisation safety studies (Rev 3) ‐ guideline‐good‐pharmacovigilance‐practices‐gvp‐module‐viii‐post‐authorisation‐safety‐studies‐rev‐3_en.pdf. https://www.ema.europa.eu/documents/scientific‐guideline/guideline‐good‐pharmacovigilance‐practices‐gvp‐module‐viii‐post‐authorisation‐safety‐studies‐rev‐3_en.pdf. Accessed October 25, 2018.

[58] Section 4.5: Interaction with other medicinal products and other forms of interaction ‐ presentation‐section‐45‐interaction‐other‐medicinal‐products‐other‐forms‐interaction_en.pdf. https://www.ema.europa.eu/documents/presentation/presentation‐section‐45‐interaction‐other‐medicinal‐products‐other‐forms‐interaction_en.pdf. Accessed October 25, 2018.

[59] Nissen T , Wynn R . The clinical case report: a review of its merits and limitations. BMC Res Notes. 2014;7:264. 24758689 10.1186/1756-0500-7-264 PMC4001358

[60] Egan G , Hughes CA , Ackman ML . Drug interactions between antiplatelet or novel oral anticoagulant medications and antiretroviral medications. Ann Pharmacother. 2014;48(6):734‐740. 24615627 10.1177/1060028014523115

[61] Strandell J , Wahlin S . Pharmacodynamic and pharmacokinetic drug interactions reported to VigiBase, the WHO global individual case safety report database. Eur J Clin Pharmacol. 2011;67(6):633‐641. 21253716 10.1007/s00228-010-0979-y

[62] UMC|Know more about VigiBase. https://www.who‐umc.org/vigibase/vigibase/know‐more‐about‐vigibase/. Accessed July 16, 2020.

[63] Strandell J , Norén GN , Hägg S . Key elements in adverse drug interaction safety signals: an assessment of individual case safety reports. Drug Saf. 2013;36(1):63‐70. 23315297 10.1007/s40264-012-0003-9

[64] Lindquist M . VigiBase, the WHO Global ICSR Database System: basic facts. Drug Inf J. 2008;42(5):409‐419.

[65] Strandell J , Caster O , Bate A , Norén N , Edwards IR . Reporting patterns indicative of adverse drug interactions: a systematic evaluation in VigiBase. Drug Saf. 2011;34(3):253‐266. 21332249 10.2165/11586990-000000000-00000

